# The Tumor Metabolite 5′-Deoxy-5′Methylthioadenosine (MTA) Inhibits Maturation and T Cell-Stimulating Capacity of Dendritic Cells

**DOI:** 10.3390/cells13242114

**Published:** 2024-12-20

**Authors:** Christina Brummer, Katrin Singer, Frederik Henrich, Katrin Peter, Carolin Strobl, Bernadette Neueder, Christina Bruss, Kathrin Renner, Tobias Pukrop, Wolfgang Herr, Michael Aigner, Marina Kreutz

**Affiliations:** 1 Department of Internal Medicine III, University Hospital Regensburg, 93053 Regensburg, Germany; 2 Bavarian Cancer Research Center (BZKF), 91054 Erlangen, Germany; 3 Department of Internal Medicine 5, University Hospital Erlangen, 91054 Erlangen, Germany; 4 Department of Otorhinolaryngology, University Hospital Regensburg, 93053 Regensburg, Germany; 5 Comprehensive Cancer Center Eastern Bavaria (CCCO), 93053 Regensburg, Germany; 6 Center of Translational Oncology (CTO), 93053 Regensburg, Germany

**Keywords:** MTA, dendritic cells, T cell, tumor metabolism, cancer immunosurveillance, polyamine, adenosine

## Abstract

Metabolite accumulation in the tumor microenvironment fosters immune evasion and limits the efficiency of immunotherapeutic approaches. Methylthioadenosine phosphorylase (MTAP), which catalyzes the degradation of 5′-deoxy-5′methylthioadenosine (MTA), is downregulated in many cancer entities. Consequently, MTA accumulates in the microenvironment of MTAP-deficient tumors, where it is known to inhibit tumor-infiltrating T cells and NK cells. However, the impact of MTA on other intra-tumoral immune cells has not yet been fully elucidated. To study the effects of MTA on dendritic cells (DCs), human monocytes were maturated into DCs with (MTA-DC) or without MTA (co-DC) and analyzed for activation, differentiation, and T cell-stimulating capacity. MTA altered the cytokine secretion profile of monocytes and impaired their maturation into dendritic cells. MTA-DCs produced less IL-12 and showed a more immature-like phenotype characterized by decreased expression of the co-stimulatory molecules CD80, CD83, and CD86 and increased expression of the monocyte markers CD14 and CD16. Consequently, MTA reduced the capability of DCs to stimulate T cells. Mechanistically, the MTA-induced effects on monocytes and DCs were mediated by a mechanism beyond adenosine receptor signaling. These results provide new insights into how altered polyamine metabolism impairs the maturation of monocyte-derived DCs and impacts the crosstalk between T and dendritic cells.

## 1. Introduction

The approval of immunotherapeutic approaches such as immune checkpoint inhibition or adoptive cell therapy has revolutionized anti-cancer treatment and improved the prognosis for many tumor entities. However, the efficiency of cancer immunotherapy remains limited in many patients, mostly due to the nature of the tumor microenvironment (TME) [1,2]. The TME is an interconnected system of non-cellular and cellular components including tumor and immune cells, cancer-associated fibroblasts (CAFs), tumor endothelial cells (TECs), and the extracellular matrix (ECM) [3]. The complex crosstalk between these cell populations supports cancer development, progression, metastatic outgrowth, and immune evasion [4]. From a metabolic point of view, tumor and stromal cells compete with immune cells for available nutrients, leading to the deprivation of essential substrates and the accumulation of waste products [5]. This metabolic reprogramming fosters the expansion of immunosuppressive cells, such as regulatory T cells (Tregs), myeloid-derived suppressor cells (MDSCs), and tumor-associated macrophages (TAMs), while blunting the activity of anti-tumoral immune cells, including dendritic cells (DCs), CD4-positive T helper cells (Th1), CD8-positive T cells (CD8+ T), natural killer (NK) cells, and M1 macrophages (M1), helping the tumor to escape from immunosurveillance [5,6].

While the impact of altered glucose and glutamine metabolism on intra-tumoral immune cells has been the subject of extensive research over recent years [5], polyamine and methionine metabolism have come into the focus of immunometabolic cancer studies only recently [7,8,9]. Many solid tumors lack or display reduced expression of the enzyme methylthioadenosine phosphorylase (MTAP), including melanomas [10], sarcomas [11,12], pancreatic cancer tumors [13,14], biliary tract tumors [15], lung tumors [16,17,18], and breast cancer tumors [19], as well as oral squamous-cell carcinoma [20], hepatocellular carcinoma [21], and endometrial carcinoma [22]. MTAP catalyzes the degradation of 5′-deoxy-5′methylthioadenosine (MTA), a byproduct of spermidine/spermine biosynthesis, into adenine, a substrate for purine biosynthesis, and methylthioribose-1-phosphate, which contributes to methionine recycling via the methionine salvage pathway [8]. Consequently, MTAP-deficient tumors accumulate MTA in the tumor microenvironment, which is associated with remodeling the intra-tumoral immune landscape towards a pro-tumoral phenotype. Tumor-derived MTA activates CAFs, induces matrix metalloproteinases (MMPs), and enhances the invasive potential of cancer cells [23,24]. Furthermore, MTA suppresses T cell proliferation and activation and effector functions, drives T cell exhaustion, and inhibits NK cell-mediated cytotoxicity, both in vitro [25,26] and in vivo [27,28]. Chang et al. have shown that MTAP-deficiency-induced reprogramming of the intra-tumoral immune cell composition promoted tumor progression and immune evasion in tumor-bearing mice [27]. These results indicate that the tumor metabolite MTA seems to be a powerful negative modulator of anti-tumoral immune defense. However, the exact mechanisms by which MTA impairs T cells, as well as its effects on other immune cells such as monocytes or dendritic (DCs), have not yet been elucidated.

In this study, we analyzed the effects of MTA on human monocytes and their maturation into DCs in vitro. We show that MTA alters the cytokine secretion profile and phenotype of monocytes. Furthermore, MTA impairs the maturation, IL-12 production, and T cell-stimulating capacity of monocyte-derived DCs. MTA-induced effects were mediated by a mechanism beyond adenosine receptor signaling. These findings provide new insights into the immunometabolic crosstalk between T cells and DCs and foster understanding of how altered polyamine metabolism might contribute to an immunosuppressive tumor microenvironment in MTAP-deficient tumors.

## 2. Materials and Methods

### 2.1. Isolation and Culture of Monocytes

Monocytes were isolated by leukapheresis from healthy donors, followed by density gradient centrifugation over Ficoll/Hypaque and separation by countercurrent centrifugation (J6M-E centrifuge; Beckmann, Munich, Germany). Monocyte purity was ≥85% as determined by CD14 expression. Isolated monocytes were cultured for 24–48 h in RPMI-1640 supplemented with 2% human AB-serum (PAN Biotech, Aidenbach, Germany), 2 mmol/L L-glutamine (Biochrom, Berlin, Germany), 50 U/mL penicillin, and 50 µg/mL streptomycin (both from Gibco, Karlsruhe, Germany). To minimize the effects of donor variation, all experiments were performed with monocytes from at least 3 different healthy donors.

### 2.2. Generation of Dendritic Cells

For the generation of monocyte-derived DCs, monocytes were cultured at a density of 1 × 10^6^ cells/mL in RPMI-1640 supplemented with 10% fetal calf serum (PAA, Cölbe, Germany), 2 mmol/L L-glutamine, 50 U/mL penicillin, 50 µg/mL streptomycin (all from Gibco, Karlsruhe, Germany), 144 U/mL IL-4 (Peprotech, Hamburg, Germany), and 225 U/mL GM-CSF (Bayer Healthcare, Leverkusen, Germany). Immature DCs (iDCs), as well as mature DCs (mDCs), were generated over a period of 7 days. To obtain mDCs, the cells were stimulated with 10 ng/mL lipopolysaccharide (LPS, Alexis, Grünberg, Germany) from day 5 to day 7 according to the protocol of Romani et al. [29]. To evaluate the effect of MTA on DCs, MTA (Sigma-Aldrich, Saint Louis, MO, USA) was added to the cultures at different concentrations over the whole period of differentiation.

For the generation of DCs from peripheral mononuclear cells (pMNCs), pMNCs were cultured at a density of 4.0 × 10^6^ cells/mL in RPMI-1640 supplemented with 2% AB serum, L-glutamine (2 mmol/L), 50 U/mL penicillin, 50 µg/mL streptomycin (all from Gibco, Karlsruhe, Germany), 144 U/mL IL-4 (Peprotech, Hamburg, Germany), and 225 U/mL GM-CSF (Bayer Healthcare, Leverkusen, Germany) in Nunc UpCell plates (ThermoFisher Scientific, Waltham, MA, USA). The cells were initially cultured for 24 h before cells were stimulated with 10 ng/mL lipopolysaccharide (LPS, Alexis, Grünberg, Germany) to induce DC maturation. To evaluate the effects of MTA (150 µM) or the PRMT5 inhibitor EPZ-015666 (5 or 10 µM, Sigma Aldrich, Saint Louis, MO, USA) on maturation, the respective compounds were added to the culture on day 0 for the whole culture period of 72 h. DMSO-treated cells were used as a control.

### 2.3. Determination of Cytokines in Cell Culture Supernatants

For the generation of monocyte supernatants, cells were incubated in 12-well cell culture plates at a density of 1 × 10^6^ cells/mL in the presence of rising concentrations of 5′-deoxy-5′methylthioadenosine (MTA) with or without addition of the A1 antagonist (A1i) 8-Cyclopentyl-1,3Dipropylxanthin (CPCPX), the A2a antagonist (A2Ai) 8-3-Chlorostyryl-coffeine (CSC), the A2B antagonist (A2Bi) alloxazin, or the A3 antagonist (A3) MRS1292 (all purchased from Sigma-Aldrich, Saint Louis, MO, USA). After 24 h, supernatants were harvested, filtered, and stored at −20 °C.

For the generation of DC supernatants, iDCs or mDCs were harvested on day 7 and further incubated in a 12-well cell culture plate at a density of 1 × 10^6^ cells/mL. After 24 h, supernatants were harvested, filtered, and stored at −20 °C. Analysis of cytokines (TNF, IL-6, IL-10, IL-12, and IL-1β) in monocyte and DC supernatants was performed by using commercially available enzyme-linked immunosorbent assays (ELISA; R&D Systems, Minneapolis, MN, USA).

### 2.4. Determination of Intracellular cAMP Levels

For the analysis of intracellular cyclic adenosine monophosphate (cAMP) levels, monocytes were lysed by the addition of 0.1 M HCl, incubated on ice for 20 min, and centrifugated. Intracellular cAMP levels were measured using the cAMP direct immunoassay kit (BioVision, Mountain View, CA, USA) according to the manufacturer’s instructions.

### 2.5. Analysis of Viability by Annexin-V/7-AAD Staining

For the analysis of cell viability, monocytes were seeded at a concentration of 1 × 10^6^ cells/mL in hydrophobic Teflon bags with or without increasing concentrations of MTA. After 48 h, cells were harvested, washed with PBS, counted, and stained with Annexin-V and 7-AAD (both from BD Biosciences, San Jose, CA, USA) according to the manufacturer’s instructions. Flow cytometric analyses were performed on a FACSCalibur (BD, Franklin Lakes, NJ, USA) using BD CellQuestPro 5.1 for data acquisition and analysis.

### 2.6. Determination of Surface Marker Expression by Flow Cytometry

For the determination of surface antigen expression, iDCs and mDCs were harvested on day 7. Cells were washed twice with cold phosphate buffer saline (PBS; GE Healthcare, Solingen, Germany) and stained with the following fluorescein isothiocyanate (FITC)- or phycoerythrin (PE)-conjugated monoclonal antibodies: anti-CD83 (from Beckman Coulter, Marseille, France), anti-CD1a, anti-CD14, anti-CD16, anti-CD80, anti-HLA-DR, and anti-CD86, with IgG as an isotype control (all from BD, Franklin Lakes, NJ, USA). For adenosine receptor staining, MNCs were stained with an A2A receptor antibody (from Chemicon, Billerica, MA, USA). As a secondary antibody, a FITC-labeled anti-rabbit IgG from Sigma-Aldrich (St. Louis, MO, USA) was used. Flow cytometric analyses were performed on a FACSCalibur or FACS Canto II (BD, Franklin Lakes, NJ, USA) using BD CellQuestPro 5.1 or FACSDiva 9.0 for data acquisition. Antigen expression was calculated by subtracting the median of the isotype control from the median of the specific staining.

### 2.7. Mixed Lymphocyte Reaction (MLR)

T lymphocytes were isolated by leukapheresis from healthy donors, followed by density gradient centrifugation over Ficoll/Hypaque and separation by countercurrent centrifugation (J6M-E centrifuge; Beckmann, Munich, Germany). To assess the T cell stimulatory potential, monocyte-derived mDCs differentiated and maturated without (positive control) or with MTA (d0–7, 15 µM or 150 µM) were co-cultured at increasing ratios with allogeneic T lymphocytes for another five days (d8–12) in RPMI containing 5% AB serum, L-glutamine (2 mmol/L), penicillin (50 U/mL), and streptomycin (50 mg/mL). Co-cultured iDCs were used as a negative control. On day 12, 0.5 Ci/0.2 mL [3H]-thymidine (Amersham Pharmacia, Piscataway, NJ, USA) was added. Incorporated radioactivity was quantified after 24 h using a beta counter (Perkin Elmer, Gaithersburg, MD, USA). 3H-Thymidin is incorporated into the DNA of proliferating cells. Since T cells but not DCs proliferate, observed radioactivity is a measure for T cell proliferation.

### 2.8. Antigen Loading of DCs

For peptide loading, monocyte-derived mDCs were generated according to the protocol reported above (Section 2.2, harvested, and incubated with 30 µg/mL of the peptide of the HCMV protein pp65 (CMVpp65495-503) and 10 µg/mL of β2-Microglobulin at 37 °C and 5% CO_2_ for two hours. Every 30 min, the cell suspension was vortexed to prevent the adhesion of cells. After the incubation time was complete, the peptide-loaded DCs were washed twice with media and counted with a CASY TT cell counter (OLS Omni Life Science, Bremen, Germany).

To load DCs with protein (recombinant HCMV pp65), monocyte-derived mDCs were generated according to the protocol reported above and 10 µL/mL of pp65 suspension was added to media during maturation on day 5. On day 7, maturated mDCs were harvested, 10 µL/mL of pp65 suspension was added, and the cells were incubated at 37 °C and 5% CO_2_ for two hours. After incubation, the protein-loaded DCs were washed twice with media and counted with a CASY TT cell counter.

### 2.9. Isolation and Antigen-Specific Expansion of CD8+ T Cells

CD8+ T cells were magnetically enriched (Miltenyi Biotec, Bergisch-Gladbach, Germany) from peripheral blood mononuclear cells from healthy donors. T cell purity was ≥95% as determined by CD3+ CD8+ expression. For antigen-specific expansion, isolated CD8+ T cells (1 × 10^5^) were co-cultured with autologous protein- or peptide-loaded DCs (2 × 10^4^) in 96-well U-bottom cell culture plates in medium supplemented with IL-2 (100 U/mL, Novartis, Basel, Switzerland) for 11–14 days. Every three to four days, the medium was renewed, and every seven days, the T cells were re-stimulated with freshly maturated protein- or peptide-loaded DCs. The purity of cultivated cells was determined weekly by flow cytometry. To analyze the expansion of antigen-specific stimulated CD8+ T cells, cells were analyzed after tetramer staining (Proimmune, Oxford, UK) by flow cytometry on a FACSCanto II. Interferon-y and IL-2 (both from BD Bioscience, San Jose, CA, USA) expression in antigen-specific stimulated CD8+ T cells was measured after incubation with Monensin (Sigma Aldrich, MO, USA).

### 2.10. Immunohistochemical Staining of Adenosine Receptors

For immunohistochemistry staining, 1 × 10^6^ monocytes were plated on microscope slides, fixated, and stained for adenosine receptors using the following antibodies: anti-pan CD45 (Diatec, Oslo, Norway), isotype control (Jackson ImmunoResearch, Newmarket, UK), anti-adenosine A1 receptor, anti-adenosine A2A receptor, anti-adenosine A2B receptor, and anti-adenosine A3 receptor (all from Chemicon, Billerica, MA, USA).

### 2.11. Statistical Analysis and Visualization

Statistical analysis was performed using GraphPad Prism 10 (GraphPad Software, La Jolla, CA, USA). Results represent at least n = 3 and are shown as the mean plus the standard deviation (SD) unless otherwise indicated. Data were analyzed by ANOVA (the Friedman test or Kruskal–Wallis test followed by Dunn’s post hoc test) for comparing more than two groups or the Mann–Whitney U/Wilcoxon test for comparing two groups. Significance is indicated for *p* < 0.05 (*), *p* < 0.01 (**), and *p* < 0.001 (***). Figures were created with GraphPad Prism (v8 and v10, GraphPad Software, La Jolla, CA, USA) or Microsoft Excel 2021 (Microsoft, Redmond, WA, USA). The graphical illustrations were created with Biorender (https://biorender.com). Flow cytometric analyses were performed via the FlowJo Software (FlowJo v10.10, BD, Ashland, OR, USA).

## 3. Results

### 3.1. MTA Alters the Cytokine Secretion Profile of Monocytes

**Figure 1:**
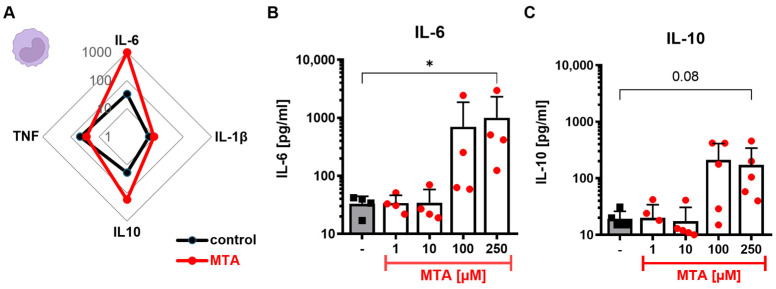
MTA alters the cytokine secretion profile of human monocytes. Unstimulated human monocytes from healthy donors were incubated for 24 h in the absence or presence of MTA. (**A**) The cytokine secretion profile of monocytes, including TNF, IL-6, IL-1β, and IL-10, incubated without (control, black) or with MTA (250 µM, red) was analyzed. Results represent the mean of n = 4–6 independent experiments. Cytokine levels are shown as log [pg/mL]. (**B**,**C**) Concentrations of (**B**) IL-6 and (**C**) IL-10 secreted by monocytes incubated without (co, black) or with increasing concentrations (1, 10, 100, or 250 µM) of MTA (red). Results represent the mean + SD of n = 4–6. Statistical analysis was performed via the Friedman test followed by the post hoc Dunn’s test. Significance is indicated for *p* < 0.05 (*).

To study the effect of MTA on monocytes, human monocytes from healthy donors were isolated, incubated for 24 h with or without increasing concentrations of MTA, and analyzed in terms of their cytokine secretion profile (Figure 1A). Unstimulated monocytes showed a concentration-dependent increase in IL-6 secretion upon incubation with 100 and 250 µM MTA (Figure 1B). Besides IL-6, IL-10 (Figure 1C) levels in cell culture supernatants also tended to increase under MTA. However, these results were not significant, and IL-1β (Figure S1A) and TNF levels (Figure S1B) remained unchanged. Since the viability (Figure S1C) of harvested monocytes remained unaffected by MTA up to a concentration of 500 µM, a cell-number-related effect could be excluded.

### 3.2. MTA Impairs the Differentiation of Monocytes to Dendritic Cells

**Figure 2:**
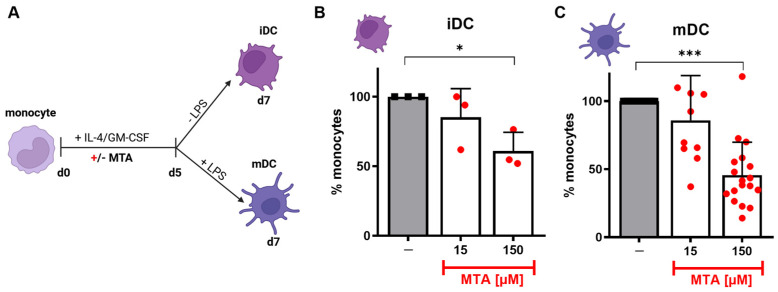
MTA alters the differentiation of monocyte-derived dendritic cells. (**A**) Monocytes were differentiated (medium + 144 U/mL IL-4 + 225 U/mL GM-CSF d0-d7) into immature (iDC, without LPS) or mature (mDC, + 10 mM LPS d5-d7) dendritic cells according to the depicted protocol in the absence (co, −, black) or presence (+, red) of 15 µM or 150 µM MTA (d0–7). Fractions of monocyte-derived (**B**) iDCs and, respectively, (**C**) mDCs harvested after seven days of differentiation are shown as a percentage of control DCs. Results represent the mean + SD of n = 3 (iDC) and n = 10–15 (mDC). Statistical analysis was performed via the Friedman test (iDC, paired) or Kruskal–Wallis test (mDC, unpaired) followed by the post hoc Dunn’s test. Significance is indicated for *p* < 0.05 (*) and *p* < 0.001 (***).

Altered cytokine secretion in monocytes is known to impact differentiation into macrophages and DCs [30]. To evaluate whether the addition of MTA has any effect on the differentiation of monocyte-derived DCs, we generated immature (iDC) and mature (mDC) dendritic cells in the absence or presence of MTA (MTA-DC, Figure 2A). MTA significantly reduced the cell yield of both iDCs (Figure 2B) and mDCs (Figure 2C) in relation to the number of seeded monocytes. Furthermore, flow cytometric analysis revealed that incubation under MTA crucially altered the morphology (Figure S2A,B) of harvested mDCs, leading to smaller and less granulated cells that exhibited phenotype patterns similar to iDCs (Supplementary Materials). These results indicated that MTA impairs DC differentiation both quantitatively and qualitatively.

### 3.3. MTA-DCs Show a More Immature-like Phenotype

**Figure 3:**
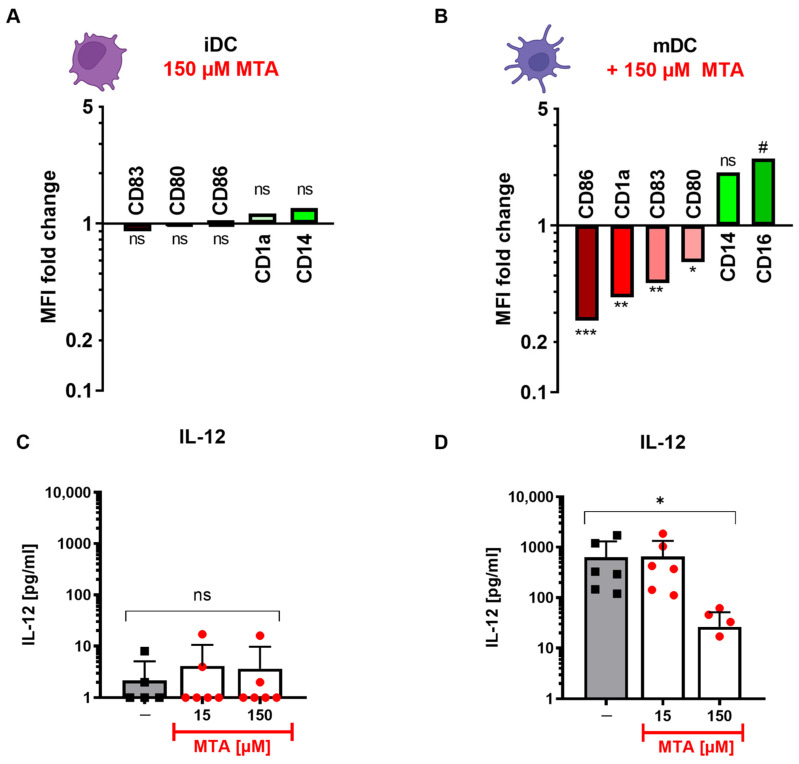
DCs maturated under MTA show a more immature phenotype and an altered cytokine production profile. (**A**,**B**) Surface marker profile of (**A**) iDCs and (**B**) mDCs differentiated with 150 µM MTA in comparison to control DCs. Results represent the mean of n = 6 (iDC) and, respectively, n = 4–17 (mDC) and are shown as MFI fold expression in relation to control DCs (set as 1) differentiated without MTA. Increased expression is depicted in green, and decreased in red. Surface marker expression with and without MTA was compared via the Wilcoxon test (iDC, paired) and Mann–Whitney U test (mDC, unpaired). Significance is indicated for *p* < 0.05 (*), *p* < 0.01 (**), *p* < 0.001 (***); # represents *p* = 0.05, ns = not significant. (**C**,**D**) Concentrations of IL-12 secreted by iDCs (**C**) and mDCs (**D**) incubated without (co, black) or with (red) increasing concentrations (15 and 150 µM) of MTA for 7 days. Results represent the mean + SD of n = 6. Statistical analysis was performed via the Friedman test followed by the post hoc Dunn’s test. Significance is indicated for *p* < 0.05 (*).

To further evaluate the impact of MTA on DCs, we analyzed the phenotypes of iDCs and mDCs differentiated in the presence (MTA-DCs) or absence (co-DCs) of MTA. For this purpose, monocytes were differentiated and maturated into DCs with or without 150 µM MTA. After seven days, co-DCs and MTA-DCs were stained for surface markers and analyzed using flow cytometry (Figure 3). MTA did not significantly alter the expression of surface antigens in iDCs (Figure 3A). In contrast, the surface marker profile of mDCs was significantly changed by MTA in comparison to control cells (Figure 3B and Figure S3A). MTA-mDCs showed a decreased expression of CD1a, as well as the co-stimulatory molecules CD80, CD83, and CD86, whereas the expression of CD16 and CD14 was increased (Figure 3B and Figure S3A). While CD14 and CD16 are known as monocyte markers, CD1a, CD80, CD83, and CD86 are established markers for DC maturation. Thus, these results indicate that MTA impairs the maturation of DCs and induces a switch towards an immature-like phenotype. MTA-induced surface marker reprogramming was accompanied by an altered cytokine secretion profile in mDCs but not iDCs (Figure 3C,D). While IL-12 (Figure 3C) and IL-10 (Figure S3B) production remained largely unchanged in iDCs, the potential of mDCs (Figure 3D to secrete pro-inflammatory cytokines such as IL-12 was significantly reduced under MTA. In contrast, IL-10 levels were unaffected (Figure S3C). These observed MTA-induced effects on surface marker expression and cytokine secretion of mDCs indicated that MTA might impact the co-stimulatory ability of mDCs.

### 3.4. MTA Impairs the T Cell-Stimulation Capacity of DCs

**Figure 4:**
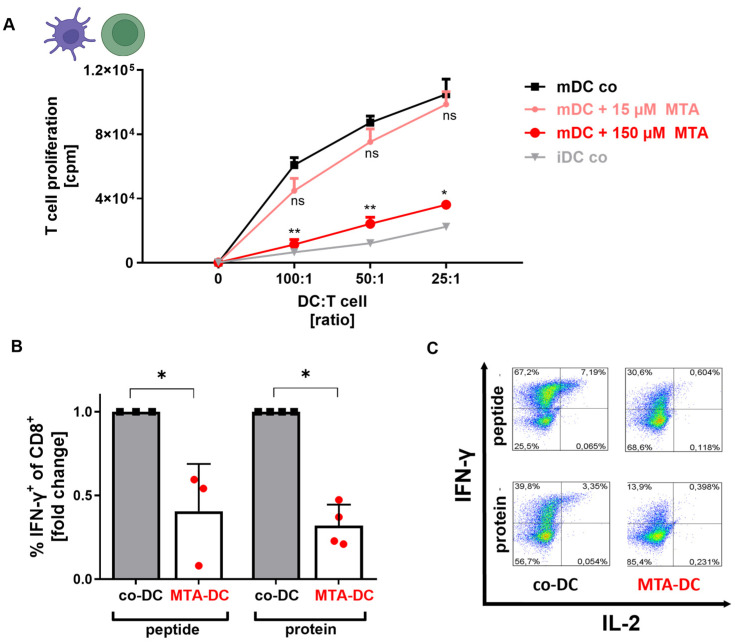
MTA impairs the T cell stimulation capacity of DCs. (**A**) mDCs were maturated from human monocytes without (co, black) or with (red) the addition of 15 or 150 µM MTA (d0–7). After maturation, mDCs were co-cultured in fixed ratios (100:1, 50:1, or 25:1) with allogeneic T cells for 5 days in a mixed lymphocyte reaction. On day 12, _3_H-Thymidin was added. Radioactivity, as a marker of T cell proliferation, was measured on d13. Results represent the mean + SD of n = 5 healthy donors. Comparisons between mDC MTA and control groups were performed via the Friedman multiple comparison test and the post hoc Dunn’s test. Significance is indicated for *p* < 0.05 (*) and *p* < 0.01 (**). (**B**,**C**) Peptide-loaded (CMVpp65_495–503_) and protein-loaded (CMVpp65) mDCs maturated without (co-DC) or with 150 µM MTA (MTA-DC) were co-cultured with autologous CD8+ T lymphocytes (ratio 5:1) from the same donor for 11 days. On day 7, CD8+ T cells were restimulated with freshly maturated peptide- or protein-loaded mDCs. On day 11, the fraction of positive antigen-specific (peptid or protein) CD8+ T cells was analyzed using flow cytometry. Unstimulated CD8+ T cells were used as the negative control. (**B**) INF-y-positive antigen-specific CD8 T cells stimulated by MTA-DCs are shown as fold changes of CD8+ T cells stimulated by co-DCs (=1). The mean + SD of n = 3–4 is shown. Co-DC and MTA-DC groups were statistically compared via the Mann–Whitney U test. Significance is indicated for *p* < 0.05 (*), ns = not significant. (**C**) Antigen-specific CD8+ T cells stimulated by co-DCs or MTA-DCs were analyzed using flow cytometry regarding INF-y and IL-2. One representative experiment is shown.

To test whether MTA impacts the T cell-inducing capacity of mDCs, we next performed a mixed lymphocyte reaction of T lymphocytes and DCs differentiated and maturated with or without MTA (Figure S4A). Control mDCs induced strong proliferation of T cells (positive control, Figure 4A). The administration of 15 µM MTA resulted in a slight but non-significant decrease in proliferation, whereas mDC maturated under 150 µM MTA lost the capacity to stimulate T cell proliferation almost to the level of iDCs used as a negative control (Figure 4). These results indicate that MTA impairs the capacity of DCs to stimulate T cells and confirm the already described inhibitory effect of MTA on the surface expression of co-stimulatory molecules on the functional level.

To further evaluate the impact of MTA on the functional stimulation capacity of DCs, mDCs maturated with (150 µM, MTA-DC) or without MTA (co-DC) were loaded with an antigen and co-cultured with freshly isolated autologous CD8+ T cells (Figure S4B). The immunogenic peptide of the HCMV protein pp65 (CMVpp65_495–503_) or the complete protein (pp65) was used as the antigen. Both co-DCs and MTA-DCs stimulated antigen-specific expansion of CTLs at comparable percentages and no difference was observed between peptide- and protein-loaded DCs (Figure S4C). This indicates that MTA-DCs are still able to stimulate antigen-specific CTLs and that the primary response of DCs as antigen-presenting cells (APCs) is not altered by MTA.

To assess the impact of MTA on the capability of DCs to induce T effector functions, we next measured IFN-γ production in CD8+ T cells stimulated with peptide- and protein-loaded co-DCs or MTA-DCs (Figure 4B). MTA-DCs induced significantly reduced IFN-γ expression in antigen-specific CD8+ T cells (Figure 4B). Besides IFN-γ, CTL also displayed reduced IL-2 production when stimulated by MTA-DCs (Figure 4C). These results indicate that MTA-DCs display an impaired capability to stimulate effector functions in antigen-specific expanded CD8+ T cells.

### 3.5. MTA-Induced Effects on Monocytes and DCs Are Mediated by a Mechanism Beyond Adenosine Receptor Agonism

**Figure 5:**
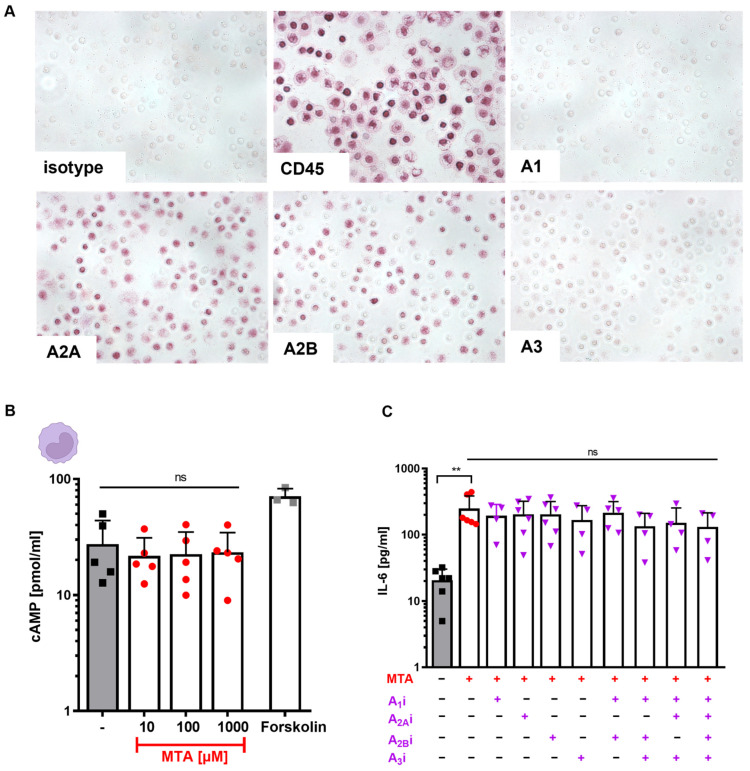
MTA-induced effects on monocytes are mediated by a mechanism beyond adenosine receptor signaling. (**A**) Immunohistochemical staining of adenosine receptors A1, A2A, A2B, and A3 on monocytes. Staining for CD45 and isotype is shown as the positive and, respectively, negative control. One representative experiment is shown. (**B**) Monocytes were incubated with or without 10, 100, or 1000 µM MTA for 60 min and lysed. Intracellular cAMP levels were measured using an immunoassay kit. Forskolin (50 µM) was used as a positive control. (**C**) Monocytes were incubated with (+) or without (−) 250 µM MTA, the A1 antagonist (A1i) 8-Cyclopentyl-1,3Dipropylxanthin (CPCPX), the A2a antagonist (A2Ai) 8-3-Chlorostyryl-coffeine (CSC), the A2B antagonist (A2Bi) alloxazin, or the A3 antagonist (A3) MRS1292 for 20 h. IL-6 concentrations of cell culture supernatants were measured by ELISA. Results represent the mean of n = 4–6 and are shown as the mean + SD. Significance was determined using one-way ANOVA and post hoc Dunn’s multiple comparisons tests (** *p* < 0.01, ns = not significant).

We next analyzed the signaling cascade by which MTA impacts monocytes and DCs. As an adenosine analogue, MTA binds to adenosine receptors [31,32]. Since adenosine receptor signaling has also been shown to play an important role in regulating immune cell functions [33,34], we hypothesized that MTA might impact the differentiation and cytokine production of monocytes through adenosine receptor agonism. To evaluate this hypothesis, we first analyzed monocytes (Figure 5A) and DCs (Figure S5A) regarding their expression of the four adenosine receptors (ADOR) A1R, A2AR, A2BR, and A3R (Figure 5A). Immunohistochemical staining (IHC) showed that both monocytes and DCs express all four ADOR receptors, mostly A2AR and A2BR (Figure 5A and Figure S5A). To confirm expression on the cell surface, complementary adenosine receptor staining of MNCs by flow cytometry was performed (Figure S5B). In line with results obtained by IHC, the staining of myeloid cells for A2AR was strongly positive (Figure S5B).

ADOR binding leads to activation (A2A, A2B) or inhibition (A1, A3) of adenylate cyclase, resulting in an increase (A2A, A2B) or decrease (A1, A3) in intracellular cAMP levels [33,34]. However, incubation of monocytes with increasing levels of MTA did not alter intracellular cAMP levels (Figure 5B). Next, monocytes were incubated with 250 µM MTA in the presence or absence of the A1R antagonist (A1i) 8-Cyclopentyl-1,3Dipropylxanthin (CPCPX), the A2AR antagonist (A2Ai) 8-3-Chlorostyryl-coffeine (CSC), the A2BR antagonist (A2Bi) alloxazine, or the A3R antagonist (A3) MRS1292 for 20 h (Figure 5C). While MTA alone induced IL-6 production, adenosine receptor blocking could not alleviate the MTA-induced effect on IL-6 production. Thus, these data suggest that MTA impairs monocytes by a mechanism beyond adenosine receptor signaling.

### 3.6. MTA-Induced Effects on DC Maturation Can Be Partly Reproduced by PRMT5 Inhibition

**Figure 6:**
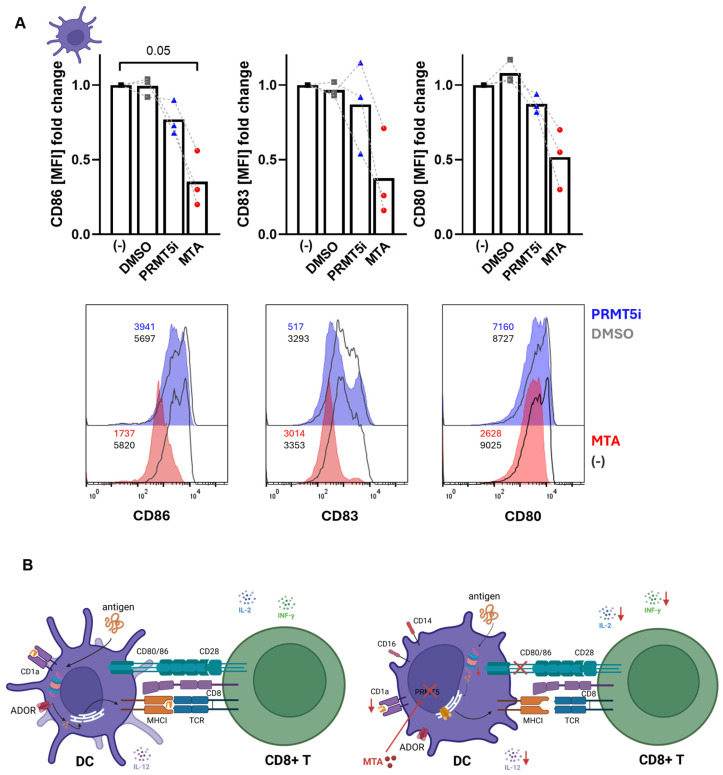
MTA-induced effects on DC maturation can be partly reproduced by PRMT5 inhibition. (**A**) Surface marker profiles of DCs differentiated from MNCs in the presence of 150 µM MTA or the PRMT5 inhibitor EPZ-0015666 (10 µM) in comparison to control and DMSO-treated DCs were analyzed by flow cytometry. Results represent the mean of n = 3. Surface markers are shown as the MFI fold expression relative to control DCs. Histogram overlays of one example donor are shown. Significance was determined using one-way ANOVA and post hoc Dunnett’s multiple comparisons tests (* *p* < 0.05). (**B**) Graphical illustration of MTA-induced effects on the crosstalk between DCs and CD8+ T cells. Red arrows indicate downregulation by MTA. The figure was created with BioRender.

MTA is known to inhibit protein arginine methyltransferases (PRMTs), especially PRMT5, which catalyzes arginine demethylation and plays an important role in cell cycle regulation [35,36,37]. To test whether MTA-induced effects on DC maturation are mediated by PRMT5 inhibition, DCs were generated from peripheral blood mononuclear cells (pMNCs) in the absence or presence of MTA (150 µM) or the PMRT5 inhibitor (PRMT5i) EPZ-015666 (10 µM) and analyzed by flow cytometry regarding their surface marker profile (Figure 6A). As shown for monocyte-derived mDCs, MTA induced a phenotype switch of DCs characterized by reduced CD86, CD83, and CD80 expression. The impact of MTA on CD86, but not CD83 or CD80, could be reproduced by treatment with the PRMT5i to some extent (Figure 6A). These data indicate that MTA is cell-permeable and MTA-induced effects on DCs might be partly mediated by PRMT5 inhibition (Figure 6B).

## 4. Discussion

Targeting cancer metabolism has been discussed as a treatment option to enhance the efficiency of immunotherapy for years [38]. However, broad inter- and intra-tumoral heterogeneity leads to a wide-ranging diversity of immunometabolic TME landscapes [39]. This challenges metabolic therapy approaches and raises the urgent need for further research to identify new targets [40]. In this study, we demonstrate that the tumor metabolite MTA alters cytokine production in human monocytes and impairs the maturation and T cell stimulation capacity of DCs. While the inhibitory impact of MTA on other anti-tumoral immune cells such as T, B, and NK cells has been reported before [25,26,27,28,41], to our knowledge, we are the first to describe MTA-induced immunomodulatory effects on human monocytes and DCs. These results provide new insights into the mechanisms by which MTAP-deficient tumors might remodel the tumor microenvironment and promote immune evasion.

In the context of anti-cancer immunity, monocytes can either exert pro- or anti-tumoral effects, depending on their phenotype and cytokine production profile [42]. In many cancer entities, tumor-derived modulators such as lactic acid impair the pro-inflammatory activity of monocytes to escape immunosurveillance [43,44]. Here, we show that MTA selectively induces the production of IL-6 and IL-10 but does not alter other cytokines or the viability of monocytes in vitro. Since intra-tumoral IL-6/IL-10-producing monocytes are known to exert immunosuppressive functions and foster immune evasion [42], this indicates that the metabolite MTA might contribute to a phenotype shift of monocytes from anti-tumoral towards pro-tumoral in the TME. To confirm this hypothesis, further in vivo studies investigating the impact of MTA in tumor mouse models are needed.

Besides on monocytes, we also observed MTA-induced effects on the maturation and activation of DCs. As APCs, DCs induce tumor-specific T cell responses and therefore play an important role in cancer immunosurveillance [45]. Monocyte-derived iDCs reside in the interstitial tissue and epidermis to sample the surrounding environment for pathogens [46]. Recognized antigens, including tumor-associated antigens, are phagocytosed, processed, and presented on the cell surface by MHC molecules [47]. Antigen-loaded mDCs present processed antigens to T cells and initiate an adaptive immune response by upregulating co-stimulatory surface molecules and secreting pro-inflammatory cytokines [47]. Tumor-infiltrating DCs are known to exert an impeded ability to take up and present antigens [45]. Besides tumor cell-derived cytokines, growth factors, and chemokines [48,49], intra-tumoral metabolites such as lactate, as well as IL-6 and M-CSF, have been shown to impair DC function [43,50,51,52]. In this study, we show that the tumor metabolite MTA contributes to the inhibition of DC differentiation and function. MTA-DCs showed a more immature-like phenotype, characterized by increased expression of monocyte markers such as CD14 and CD16, as well as decreased expression of the co-stimulatory molecules CD80, CD86, and CD83, which are needed for cross-stimulation of T cells. Furthermore, MTA-DCs lost CD1a expression. CD1 molecules are associated with the ability of DCs to produce IL-12 and polarize CD4+ T cells towards a Th1 phenotype [53]. In addition, we found significantly reduced levels of IL-12 in DCs maturated under MTA. For several tumor entities, the density of CD1a+ DCs has been shown to correlate with improved clinical outcomes [54,55,56,57]. Since CD1a+ DCs mainly present non-peptide antigens to T cells, especially glycolipids, CD1a+ DCs play a significant role in presenting tumor-associated antigens [58]. Vice versa, loss of CD1a expression indicates a TME shift from an anti-tumoral phenotype towards a pro-tumoral phenotype and is associated with reduced CD1a-mediated glycolipid-specific T cell activation [58].

Indeed, MTA-DCs displayed a reduced capability to stimulate T cells in a mixed lymphocyte reaction in our study. Moreover, experiments with antigen-loaded DCs revealed that MTA-DCs maintain the capability to stimulate the expansion of antigen-specific CD8+ T cells, but show a reduced ability to induce effector functions, including IFN-γ and IL-2 production. This indicates that MTA-DCs display a sustained primary response but are impaired in their secondary response. In contrast to the classical presentation of internalized processed antigens on MHC-II molecules, antigen presentation via MHC I, e.g., of tumor-associated antigens, relies on cross-presentation via upregulated co-receptors and the secretion of pro-inflammatory cytokines [59]. Along these lines, we found significantly reduced expression of the co-stimulatory molecules CD80/86 and pro-inflammatory cytokine IL-12 in MTA-DCs. Furthermore, CD83 expression was reduced, which is known to be an important co-factor for CD86 and MHC-II upregulation [60]. MTA-induced suppression of tumor-infiltrating CD8+ T cells has been demonstrated before, both in vitro and in vivo [25,27,28,35,41]. In line with our results, Chang et al. recently reported that MTAP deficiency resulted in remodeling of the intra-tumoral immune landscape in tumor-bearing mice characterized by decreased tumor-infiltrating T cells [27]. Vice versa, polyamine blockade increased the number of intra-tumoral T cells and enhanced anti-tumor immunity in another mouse model [61]. Preliminary data about MTA-induced T cell inhibition mostly report direct intracellular effects as underlying mechanisms, including reprogramming of chromatin accessibility [28] and impairment of protein methylation [25,35]. Here, we show that MTA-induced effects on T cells are further indirectly mediated by impaired stimulation via DCs via reduced expression of co-stimulatory surface markers on the one hand and reduced IL-12 secretion on the other hand. Interestingly and in contrast to previously reported MTA-induced direct effects on T cells [35,36], MTA-induced effects on monocytes and DCs were mediated by a mechanism beyond adenosine receptor signaling. Recent studies have shown that MTA-mediated effects on T cells are partly mediated by inhibition of the protein arginine methyltransferase 5 (PRMT5) [35,36]. Our results demonstrated that MTA-induced effects on DC maturation could only partly be reproduced by PRMT5 inhibition. This indicated that MTA is cell-permeable and might exert its effects on DCs mainly through intracellular mechanisms; however, its detailed mechanism of action has to be further evaluated in future studies.

In conclusion, this study shows that the tumor metabolite MTA alters cytokine production in monocytes and impairs DC maturation and T cell-stimulating capacity mediated by direct (downregulation of costimulatory surface markers) and indirect (reduced IL-12 secretion) mechanisms. These data provide new insights into the immunometabolic crosstalk between DCs and T cells. Therefore, our results add new aspects to previously reported data about MTA-induced effects on T lymphocytes by presenting an indirect mechanism of MTA-mediated T cell inhibition via an impaired DC-stimulating capacity [27]. From a clinical perspective, these results are highly relevant, since DCs are promising candidates for adoptive cell therapy approaches as target cells, vaccine carriers, or APCs for naive T cells ex or in vivo [62]. Thus, a better understanding of the influencing factors behind DC differentiation and activation in the tumor microenvironment is urgently needed. While the MTA-induced impact on other immune cells, such as NK cells, macrophages, and T cells, has been analyzed by us and several other groups before [25,26,63], to our knowledge, we are the first to report on MTA-mediated effects on monocytes and DCs.

Reports about intra-tumoral MTA levels are rare and divergent [23,64,65], but preliminary data indicate that the MTA concentrations we used in this study (150 µM) can be reached in the tumor microenvironment. Moreover, MTA concentrations between 100 and 250 µM are standard concentrations used by several other groups for studying the impact of MTA on immune cells in vitro and in vivo [35,37,63]. This suggests that the results obtained in our in vitro experiments should be transferable into the in vivo setting. However, metabolite concentrations are known to display wide-ranged intra- and intertumoral differences. Thus, to confirm our data and further evaluate the impact of MTA on intra-tumoral immune cell crosstalk, additional studies in MTAP-deficient tumor mouse models or ex vivo analyses of tumor tissues of different cancer entities are needed.

## Data Availability

The original contributions presented in this study are included in the article/Supplementary Materials. Further inquiries can be directed to the corresponding author.

## Supplementary Materials

The following supporting information can be downloaded at: https://www.mdpi.com/article/10.3390/cells13242114/s1. Supplementary Figure S1. MTA impacts the cytokine secretion but not viability of monocytes. Supplementary Figure S2. MTA alters the morphology of monocyte-derived dendritic cells. Supplementary Figure S3. MTA impacts the surface marker profile and cytokine secretion of DCs. Supplementary Figure S4. MTA reduces the capacity of mDCs to stimulate T cells. Supplementary Figure S5. MTA-induced effects on monocytes and DCs are mediated by a mechanism beyond adenosine receptor signalling.
