# Supplementary material for: The Tumor Metabolite 5′-Deoxy-5′Methylthioadenosine (MTA) Inhibits Maturation and T Cell-Stimulating Capacity of Dendritic Cells
Source: Cells. 2024 Dec 20;13(24):2114. doi: 10.3390/cells13242114 (PMC11727219; doi:10.3390/cells13242114)
Supplement: Supplementary file 1 [file cells-13-02114-s001.zip › cells-3313175-supplementary.pdf]

Supplementary Material

Supplementary Figure S1

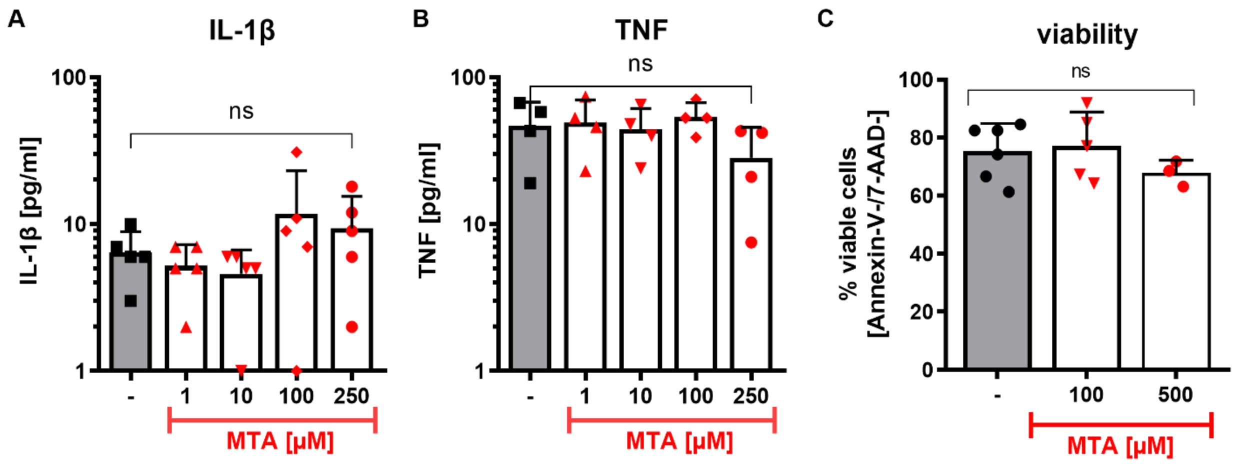

Supplementary Figure S1. MTA impacts the cytokine secretion but not viability of monocytes. Unstimulated human monocytes from healthy donors were incubated for 24 hours in the absence (co, black) or presence (red) of increasing concentrations of MTA. (A, B) Cytokine levels in cell culture supernatants were measured. (C) Cells were stained with Annexin-V/7-AAD and analysed by flow cytometry after 48 hours. Double negative cells were regarded as viable and are shown as fraction of total cells. Results represent the mean + SD of n=3-5. Statistical analysis was performed via Friedman (cytokines) or Kruskal Wallis test (viability) followed by post-hoc Dunn‘s test. Significance is indicated for $p < 0.05$ (*).

Supplementary Figure S2

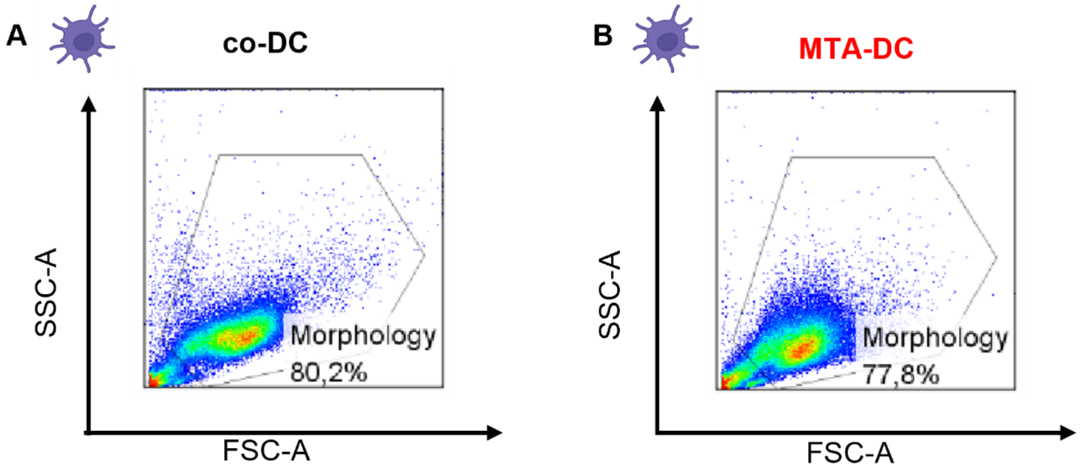

Supplementary Figure S2. MTA alters the morphology of monocyte-derived dendritic cells. Human monocytes were differentiated into mDCs in the absence (A, co-DC) or presence (B, MTA-DC) of 150 µM MTA. Morphology of harvested mDCs at d7 was analysed by flow cytometry. One representative experiment is shown.

Supplementary Figure S3

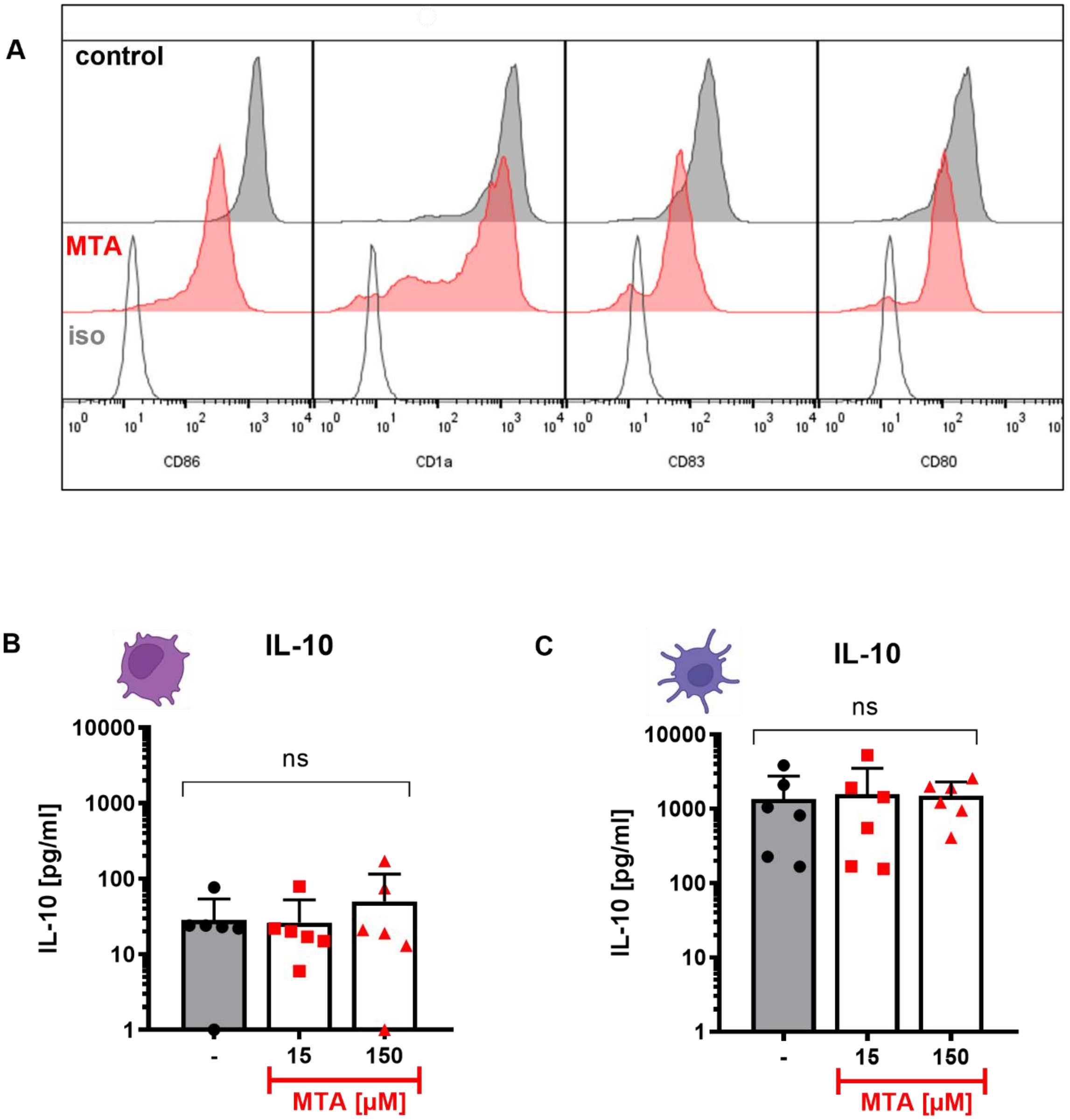

Supplementary Figure S3. MTA impacts the surface marker profile and cytokine secretion of DCs. (A) Monocytes were differentiated to mDCs in the presence (red) or absence (control, black) of 150 µM MTA. Surface marker expression of CD86, CD1a, CD83 and CD80 was analysed by flow cytometry. One representative experiment is shown. (B, C) iDCs (B) and mDCs (C) were differentiated from human monocytes in the absence (-, black) or presence (red) of increasing concentrations of MTA. IL-10 levels in cell culture supernatants were measured. Results represent the mean + SD of n=6. Statistical analysis was performed via Friedman followed by post-hoc Dunn‘s test. Significance is indicated for $p < 0.05$ (*).

Supplementary Figure S4

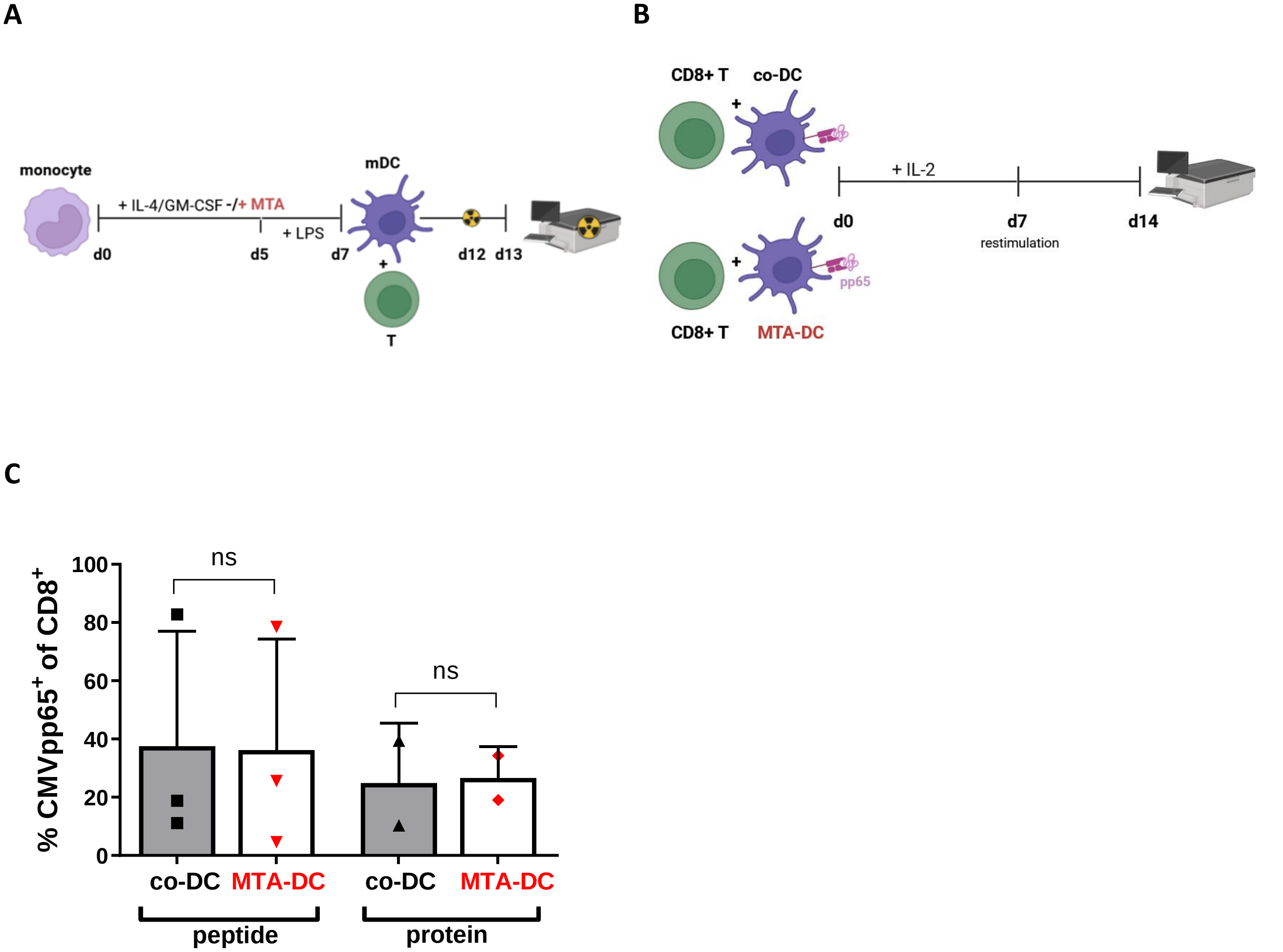

Supplementary Figure S4. MTA reduces the capacity of mDCs to stimulate T cells. (A) Experimental design of the mixed lymphocyte reaction of DCs and T cells. mDCs were maturated from human monocytes without or with addition of 15 or 150 µM MTA (d0-7). After differentiation mDCs were co-cultured in fixed ratios (100:1, 50:1, 25:1) with T cells for 5 days in a mixed lymphocyte reaction. At day 12 3H-Thymidin was added. Radioactivity as a marker of T cell proliferation was measured at d13. (B) Experimental design of antigen-specific expansion of CD8+ T cells by co-DCs vs. MTA-DCs. Peptide- (CMVpp65$_{495-503}$) or protein-loaded (CMVpp65) mDCs maturated without (co-DC) or with 150 µM MTA (MTA-DC) were co-cultured with autologous CD8+ T lymphocytes (ratio 5:1) from the same donor for 11 days. At day 7 CD8+ T cells were restimulated with freshly maturated peptide- or protein-loaded mDCs. At day 11, proliferation and cytokine production of antigen-specific (peptide or protein) CD8+ T cells was analysed by flow cytometry. (C) Fraction of antigen-specific (peptide or protein) CD8+ T cells stimulated by co-DCs or MTA-DCs. The mean + SD of n=2-3 experiments is shown.

Supplementary Figure S5

A

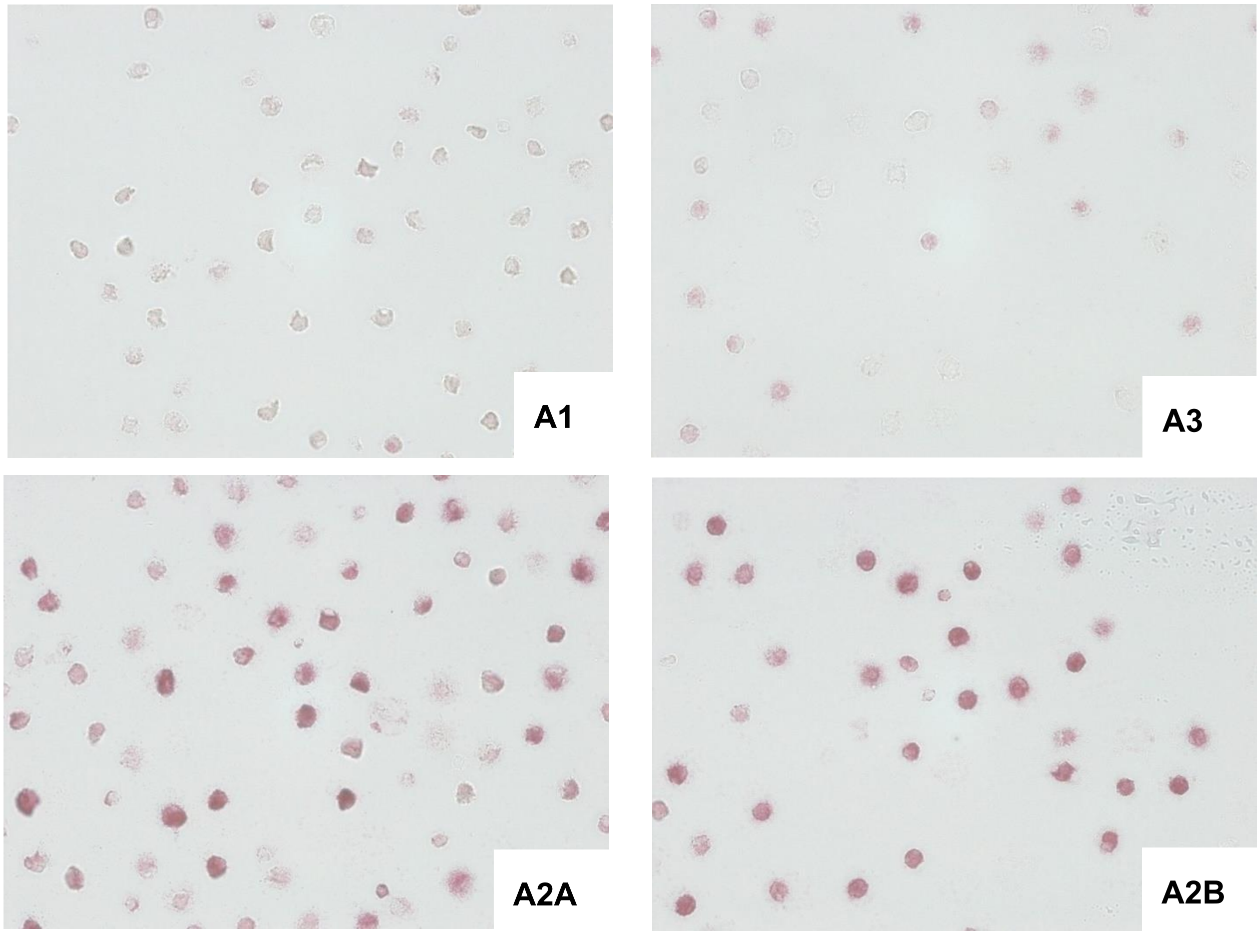

B

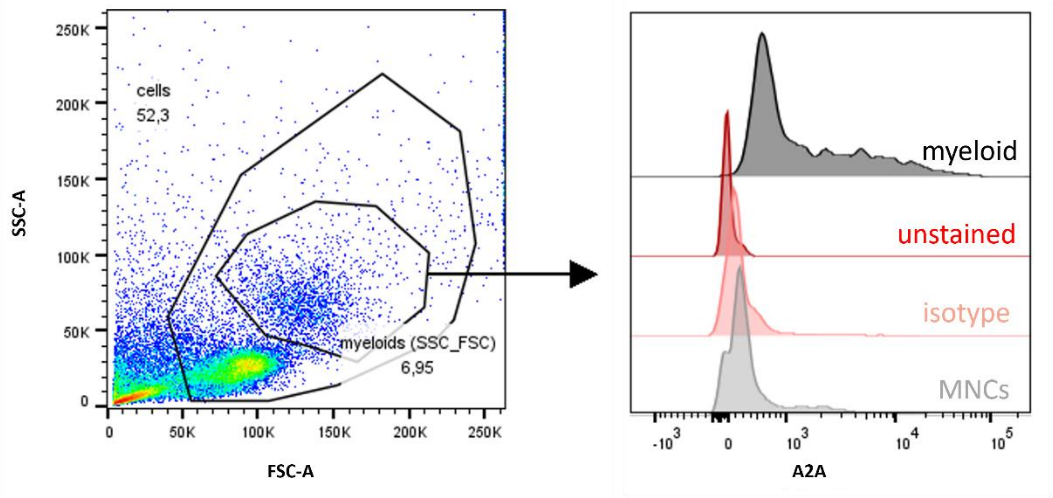

Supplementary Figure S5. MTA-induced effects on monocytes and DCs are mediated by a mechanism beyond adenosine receptor signalling. (A) Immunohistochemical staining of adenosine receptors A1, A2A, A2B and A3 on dendritic cells. Staining for isotype and CD45 (not shown) was used as positive and, respectively, negative control. One representative experiment is shown. (B) Peripheral mononuclear cells (MNCs) stained with adenosine receptor 2A (A2A) antibody were analysed by flow cytometry. While total MNCs (grey) are only slightly positive, myeloid cells (black), gated by forward (FSC-A) and side (SSC-A) scatter signal, display strong positive surface expression of A2A receptor. The FITC-labelled secondary antibody is shown as isotype (light red), unstained cells (dark red) as negative control. One representative experiment is shown.
